# Getting to grips with resilience: Toward large-scale phenotyping of this complex trait*

**DOI:** 10.3168/jdsc.2023-0434

**Published:** 2023-11-17

**Authors:** N.C. Friggens, M. Ithurbide, G. Lenoir

**Affiliations:** 1 Université Paris-Saclay, INRAE, AgroParisTech, UMR Modélisation Systémique Appliquée aux Ruminants, 91120, Palaiseau, France; 2 INRAE, Agrocampus-Ouest, PEGASE, 35590 Saint-Gilles, France; 3 GenPhySE, INRAE, ENVT, Université de Toulouse, 31326 Castanet-Tolosan, France; 4 AXIOM, 37310 Azay-sur-Indre, France

## Abstract

The capacity of animals to cope with environmental perturbations, hereafter called resilience, is an increasingly important trait. Resilience at the level of the animal is an emergent property of multiple underlying mechanisms (physiological, immunological, behavioral). This means that there is no direct measure of resilience, no easy key traits. Resilience is a latent variable that may be inferred from multivariate measures. Further, the flexibility that resilience provides is evidenced in the rate of response to, and rate of recovery from, the environmental perturbation. Thus, it requires time-series measurements. The increasing availability of on-farm precision livestock technologies, which are capable of providing time-series measures of performance and of various physiological and health biomarkers, offer the opportunity to move toward large-scale phenotyping of resilience. There have been numerous studies putting forward methods to quantify resilience. These methods can be classified as being data driven or concept driven. However, new candidate resilience proxies need to be validated. This is tricky to do because there is no direct measure of resilience, no easy gold standard measure. Per definition, good resilience will benefit the animal. Thus, the accumulated consequences of resilience can be used to evaluate resilience proxies. All other things being equal, it is expected that good resilience will be associated with a longer functional longevity (longevity adjusted for production level), with more reproductive cycles, and with fewer disease events. Recent examples of this approach of evaluating resilience proxies against the accumulated consequences of resilience are discussed. They show clearly that operational resilience proxies that are heritable and have been validated against the consequences of good resilience can be derived from on-farm time-series data. With the aim of deriving more nuanced phenotypes, there are an increasing number of studies that have taken up the challenge of attempting to statistically combine the information coming from multiple time-series measures. These studies show how multivariate time-series statistics can be used to derive more nuanced resilience phenotypes that capture some of the underlying mechanisms of resilience. In conclusion, the recent studies reviewed here have shown that operational and heritable resilience proxies exist, that they can form the basis for selection for resilience, and that more nuanced phenotypes are attainable, which will allow selection for resilience to be tailored according to prevailing environmental challenge types.

## Highlights

• Resilience is an emergent property of multiple underlying mechanisms.
• Phenotyping resilience requires time-series measurements by on-farm precision technologies.
• New resilience proxies should be validated against the accumulated consequences of resilience.
• Unpacking the underlying mechanisms is essential to better manage potential antagonisms.

## Graphical Abstract

**Figure:**
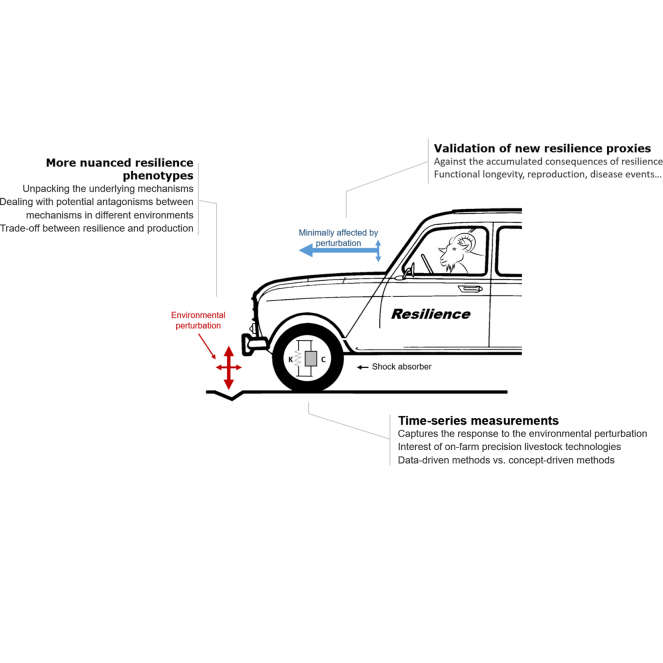
**Summary:** The resilience of an animal can be compared with the shock absorbers of a car, which buffer road bumpiness. Just as the quality of a shock absorber is measured by its ability to respond to road conditions, measuring animal resilience requires capturing the response to environmental challenges through time-series measurements. On farm precision livestock technologies offer valuable opportunities in this regard. Environmental disturbances can be diverse, and the relative importance of different underlying resilience mechanisms (depicted by K and C) can change; snow tires are useful in winter but not on sand. Understanding the mechanisms may enable us to construct more nuanced resilience phenotypes. To validate a new shock absorber, we need long-term customer feedback. Likewise, resilience indicators need to be validated against the cumulative consequences of resilience, such as functional longevity.

The capacity of animals to cope with environmental perturbations, hereafter called resilience, seems to be an increasingly important trait. It is a trait that is highly valued by farmers who in surveys refer to easy-care cows or anonymous cows (i.e., the animals that do not require attention or intervention; Spiegel et al., 2021; Christiansen et al., 2022). The increasing frequency of environmental perturbations associated with climate change and the likelihood that ruminants will be increasingly deployed in marginal environments, or fed poorer quality feeds, strongly suggest that the value of animal resilience will only increase (Yatoo et al., 2012; Tixier-Boichard et al., 2015; IPCC, 2022). However, resilience is challenging to measure.

Numerous and varied definitions of resilience have been proposed (Döring et al., 2015; Colditz and Hine, 2016; Scheffer et al., 2018; Meuwissen et al., 2019; Doeschl-Wilson et al., 2021; Friggens et al., 2022) but all agree that resilience at the level of the animal is an emergent property of multiple underlying mechanisms (physiological, immunological, behavioral) and their associated genetic determinism. This means that there is no direct measure of resilience, no easy key traits. Resilience is a latent variable that may be inferred from multivariate measures. Even though moderate heritabilities for resilience-related traits have been found (e.g., Nayeri et al., 2017; Mucha et al., 2022), this also implies that the heritable component of resilience is spread across multiple genes. A further consideration is that the panel of underlying mechanisms (and thus physiological traits) will have differing relative importance depending upon the type of environmental challenge. It is relatively easy to envisage that the major underlying mechanisms solicited when responding to a disease challenge will not be the same as those needed for coping with a nutritional challenge (Louvandini et al., 2006; Simpson et al., 2009; Doyle et al., 2011). Even without evoking such extremes, Tsartsianidou et al. (2021) found that resilience to cold weather (10°C) of animals that start producing milk in spring was under different genetic control compared with autumn and winter, exemplified by negative genetic correlations (− 0.09 to − 0.27). Likewise, Sigdel et al. (2019) found that milk yield in thermoneutral versus thermo-stressing conditions are antagonistic traits. Findings like these also suggest that the option of simply selecting animals for performance in harsh or variable environments as a means to co-select resilience may not be the most efficient way to make progress, especially in the context of genomic selection with its possibility to more precisely target underlying mechanisms (Friggens et al., 2017). Furthermore, a key question for resilience selection strategies is when should they focus on resilience to specific types of environmental challenge, for example, selecting heat-tolerant dairy cows (Nguyen et al., 2016), and when should they focus on improving general resilience (i.e., harnessing those mechanisms that are common across challenge types)?

The second feature of resilience that makes it difficult to measure is that it requires time-series measurements. The flexibility that resilience provides is evidenced in the rate of response to, and rate of recovery from, the environmental perturbation (Lough et al., 2015; Sadoul et al., 2015; Poppe et al., 2020; Ben Abdelkrim et al., 2023). Figure 1 shows an example of the individual variability in the dynamic of response and recovery. These dynamic aspects of resilience can only be captured from time-series data, and only from animals that experience an environmental perturbation. The elasticity of the system is only revealed when the system is “stretched.” Given the need for multivariate *and* time-series data, it seems at first sight that it will not be easy to phenotype resilience, at least at large scale. However, the increasing availability of on-farm precision livestock technologies that are capable of providing time-series measures of performance and of various physiological and health biomarkers offers the opportunity to move toward large-scale phenotyping of resilience. The remainder of this article discusses the approaches to measuring resilience and the challenges involved.

**Figure 1:**
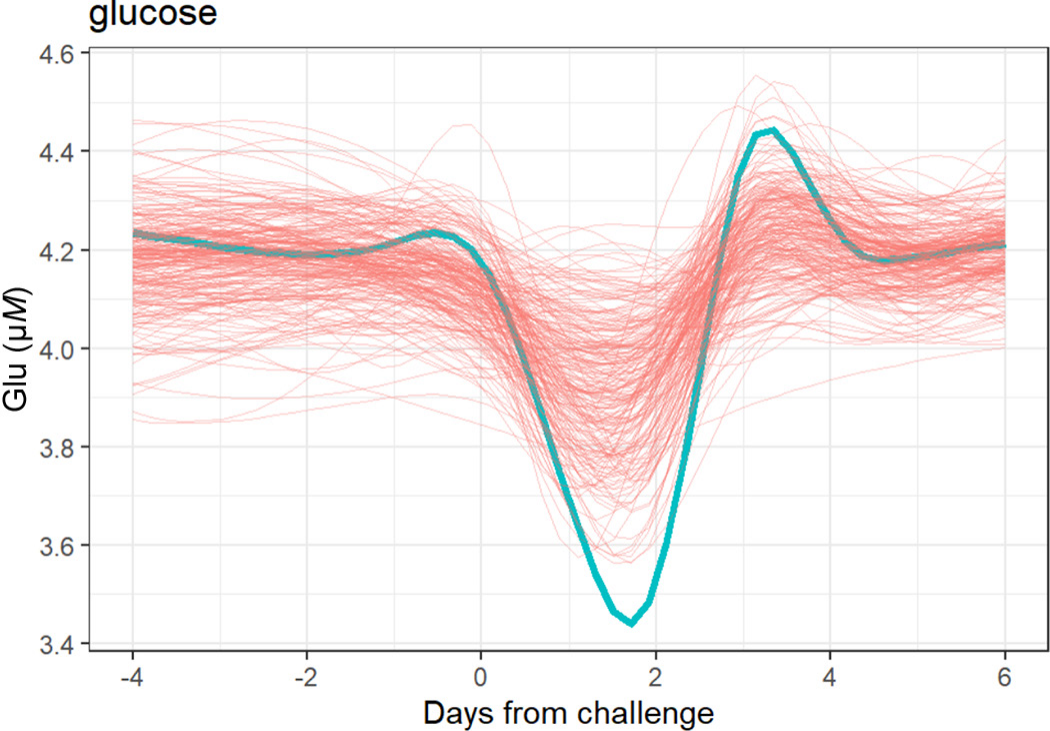
Shows an example (glucose in milk) of the individual variability in the dynamic of response to and recovery from a 2-d nutritional challenge (details in Ithurbide et al., 2023). Each line is an individual trace; an example of 1 animal is highlighted by the thick blue line. These dynamic aspects of resilience can only be captured from time-series data, and only from animals that experience an environmental perturbation; the elasticity of the system is only revealed when the system is “stretched.”

In recent years there have been numerous studies putting forward methods to quantify resilience. These methods can be classified as being data driven or concept driven, with inevitably some methods that sit in between these 2 classes. In general, all these methods seek to establish a baseline, unperturbed, time trend against which to quantify deviations from the baseline. The data-driven methods make no a priori assumptions about the baseline, and derive it directly from the data by standard smoothing methods (moving medians, splines, and so on) usually applying a high degree of stiffness to the smoothing and often giving lower weight to negative residuals in the fitting process (Codrea et al., 2011). The concept-driven methods assume an a priori functional form for the baseline, for example, a Woods curve for milk production data (Ben Abdelkrim et al., 2021) or a Gompertz function for growth data (Revilla et al., 2019) or intake (Nguyen-Ba et al., 2020). Then, deviations in the observed time-course relative to the baseline can be quantified to assess the impact of environmental perturbations. The concept-driven methods emerged partly as a way to deal with one of the shortcomings of the simpler data-driven methods, namely that when the baseline is solely derived from the data there is a tendency to underestimate longer-lasting deviations. Longer dips in the data inevitably drag the baseline down. However, these concept-driven methods impose a functional form (often a nonlinear function), which is assumed to apply to all animals in the data set, and is usually more costly to fit in terms of computing time. One interesting “in between” method class is the dynamic linear model, a particular case of so-called “state-space models.” These models can be described using 2 equations: an observation equation, relating observations and state variables, and a system equation, describing the changes of state variables over time (West and Harrison, 1997). These models allow an anticipated trajectory to be factored in. An example of a dynamic linear model being used to quantify resilience is Lenoir et al. (2022).

The approaches to characterizing the deviations in time-series data in terms of resilience mirror the above-described spectrum from data-driven to concept-driven methods. The deviations have been characterized by simply calculating the residual variance (Poppe et al., 2020) or using the turning points of spline functions to calculate amplitudes of response and time for recovery (Ben Abdelkrim et al., 2021). They have also been characterized assuming a piecewise structure to the pre-, during, and post-perturbation time-series (Friggens et al., 2016) or even explicitly assuming that resilience can be modeled using the physics analogy of a damped spring (Sadoul et al., 2015). The recent review of Taghipoor et al. (2023) describes these different models in more detail.

There is another key issue for phenotyping resilience that applies, regardless of the method used to quantify the deviations in time-series data. What is the biological meaning of these deviations? Do they actually reflect resilience? In other words, new candidate resilience proxies need to be validated. This is tricky to do because there is no direct measure of resilience, no easy gold standard measure. Another approach is needed to validate resilience proxies. The rationale for this starts by focusing on the consequences of good or bad resilience as an emergent property. As described by Friggens et al. (2022), per definition, good resilience will benefit the animal. Thus, the accumulated consequences of resilience can be used to evaluate resilience proxies. All other things being equal, it is expected that good resilience will be associated with a longer functional longevity (longevity adjusted for production level), with more reproductive cycles, and with fewer disease events (Adriaens et al., 2020; Rostellato et al., 2021; Lenoir et al., 2023). The caveat “all other things being equal” is important as it is well established that phenotypes such as functional longevity are influenced by other factors than just resilience. Indeed, it has been shown that farm level management factors impinge considerably on functional longevity and thus interfere with the calculation of farm-level resilience rankings (Adriaens et al., 2020).

There are recent examples of this approach of evaluating resilience proxies against the accumulated consequences of resilience. Poppe et al. (2020) used daily milk records to calculate the log variance of the residuals in milk yield after adjusting for the effect of the overall lactation curve, and proposed this as a resilience proxy. The data set used contained data from over 2,000 farms with more than 200,000 first-parity cows for which pedigree information was available. Accordingly, this study was able to show that there were negative genetic correlations between this simple resilience proxy and functional longevity. In other words, the greater the residual variance, the poorer was functional longevity. Lenoir et al. (2023) extended this approach by looking not at a single performance trait but rather at resource allocation. They calculated the proportion of energy intake being allocated to growth in 5,000 growing pigs, and calculated the log-squared residuals of resource allocation (relative to the linear trend in allocation with age; Figure 2). The resilience proxy was found to be heritable (h^2^ = 0.05). To test it against the accumulated consequences of resilience, Lenoir et al. (2023) calculated a robustness score that combined (lack of) mortality, vitality, and number of health events. They then showed that the proportion of animals with the high robustness score increased with increasing quartiles of the estimated breeding values for good resilience (i.e., smaller log-squared residuals). These 2 examples show clearly that operational resilience proxies, which are heritable and have been validated against the consequences of good resilience, can be derived from on-farm time-series data. The study of Lenoir et al. (2023), using the concept of resource allocation as its basis, is also a first step toward gaining a more nuanced phenotype of resilience.

**Figure 2:**
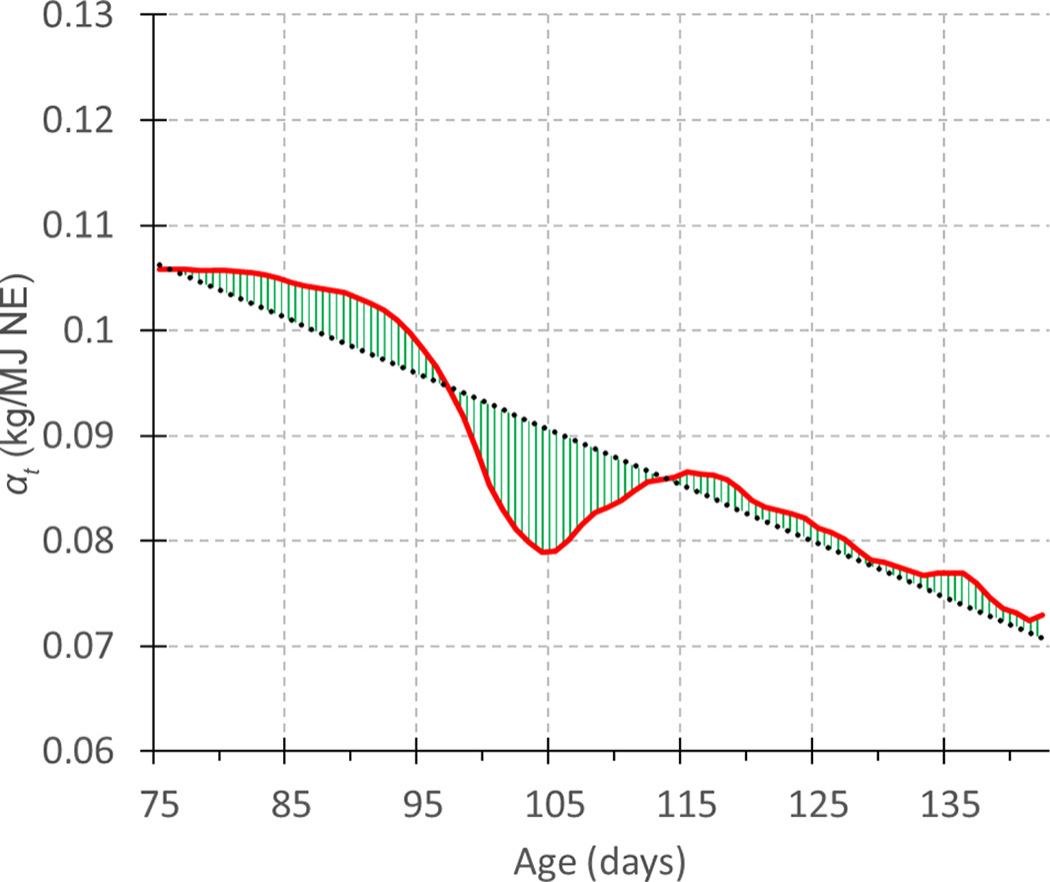
Shows an example of dynamic trajectory of the resource allocation coefficient α_t_ (net energy [NE] available that is allocated to growth) during the whole fattening period for one animal: measurements smoothed with a dynamic linear model (red line), its prediction from a random regression model (dotted line), and deviations associated to resilience (green hatched area), with details in Lenoir et al. (2023).

There are several reasons for wishing to have more nuanced phenotypes of resilience. The multivariate nature of resilience, building on multiple underlying mechanisms, implies that there will be considerable variability in the ways by which animals respond to a given challenge (Bateson and Gluckman, 2011). This in turn suggests that when faced with a different type of environmental perturbation there may be a reranking, depending upon which mechanisms are favored by different individuals. In this context, tools and measures that allow identification of the perturbation type would provide highly valuable additional information. This can be achieved using experimental perturbations of different types on the same animals, which has all the advantages of controlled conditions (nature, start, and stop times of the imposed perturbations) but is likely to be limiting in terms of numbers of animals that can be phenotyped. Alternatively, naturally occurring perturbations can be exploited if there is accompanying environmental information such as local meteorological information. In this context, studies have shown effects of weather on performance (Bunning and Wall, 2022). It can also be achieved by using animal measures. For example, on-farm monitoring of mastitis indicators such as SCC allows disease perturbations to be readily identified. Further, Garcia-Baccino et al. (2021) showed how the degree of synchrony in deviations in performance within a herd could be used to identify times when perturbations were occurring. Le et al. (2022) developed a so-called “up and down” method for detecting perturbations based on longitudinal data of intake or weight, at different scales: group, pen, or individual. These elements would contribute to an improved use of genotype-by-environment interactions approaches for identifying resilient animals (Murani et al., 2023; see also Garcia-Baccino et al., 2021).

Another reason for wanting to open the black box, to have more nuanced phenotypes, is to be able to better understand the linkages between resilience and other traits. The study of Poppe et al. (2020) found that there was a positive correlation between the resilience proxy and milk yield, indicating that higher producing animals were less resilient. Likewise, Lenoir et al. (2023) found an unfavorable genetic correlation between their resilience proxy and feed efficiency, suggesting a possible trade-off between resilience and efficiency. These results suggest that selection for improved resilience will need to take into consideration these potential trade-offs (Bouquet et al., 2022; Ghaderi Zefreh et al., 2023).

With the aim of deriving more nuanced phenotypes, an increasing number of studies have taken up the challenge of attempting to statistically combine the information coming from multiple time-series measures. Højsgaard and Friggens (2010) proposed a multivariate state-space model to capture the degree of infection associated with mastitis. This assumed that the short-term deviations in the time-series of 3 mastitis markers (SCC, conductivity, and LDH) were all reflecting changes in an underlying degree of infection. This proof of principle study was able to show that onset of, response to, and recovery from mastitis was a continuous process quite different from the traditional binary classification of mastitis as healthy versus sick. It also showed the value of the more nuanced phenotype in terms of allowing early detection, 5 d before the recorded treatment day.

Ithurbide et al. (2023) proposed another approach for exploring the resilience to a short-term nutritional challenge. In this study, 14 milk metabolites were measured daily throughout the prechallenge, challenge, and postchallenge phases for 138 dairy goats. They proposed the analytic pipeline shown in Figure 3. Central to this pipeline is a functional principal components analysis (**fPCA**) that for each metabolite captures the key dimensions of variability in the time-series trajectory. With the resulting fPCA scores, an unsupervised clustering was carried out. This found 3 clusters with significant differences in longevity between clusters. Thus, the analysis found differences in resilience phenotypes that related to differences in longevity. Given the nature of the data and the analysis, inferences about the underlying mechanisms can be made. It seems that shorter longevity was associated with goats that had more extreme lipomobilization responses to the short-term challenge and longer recoveries in the carbohydrate metabolite markers. The details of the statistical method and the results are presented in Ithurbide et al. (2023). This study shows how multivariate time-series statistics can be used to derive more nuanced resilience phenotypes. Interestingly, a supervised clustering was also carried out, using the fact that the animals in the study were daughters of bucks that were divergent on longevity. This supervised clustering on fPCA scores did not readily distinguish high versus low longevity animals, suggesting again that there is additional resilience information to be gained by seeking out resilience proxies at the level of responses to and rates of recovery from environmental perturbations.

**Figure 3:**
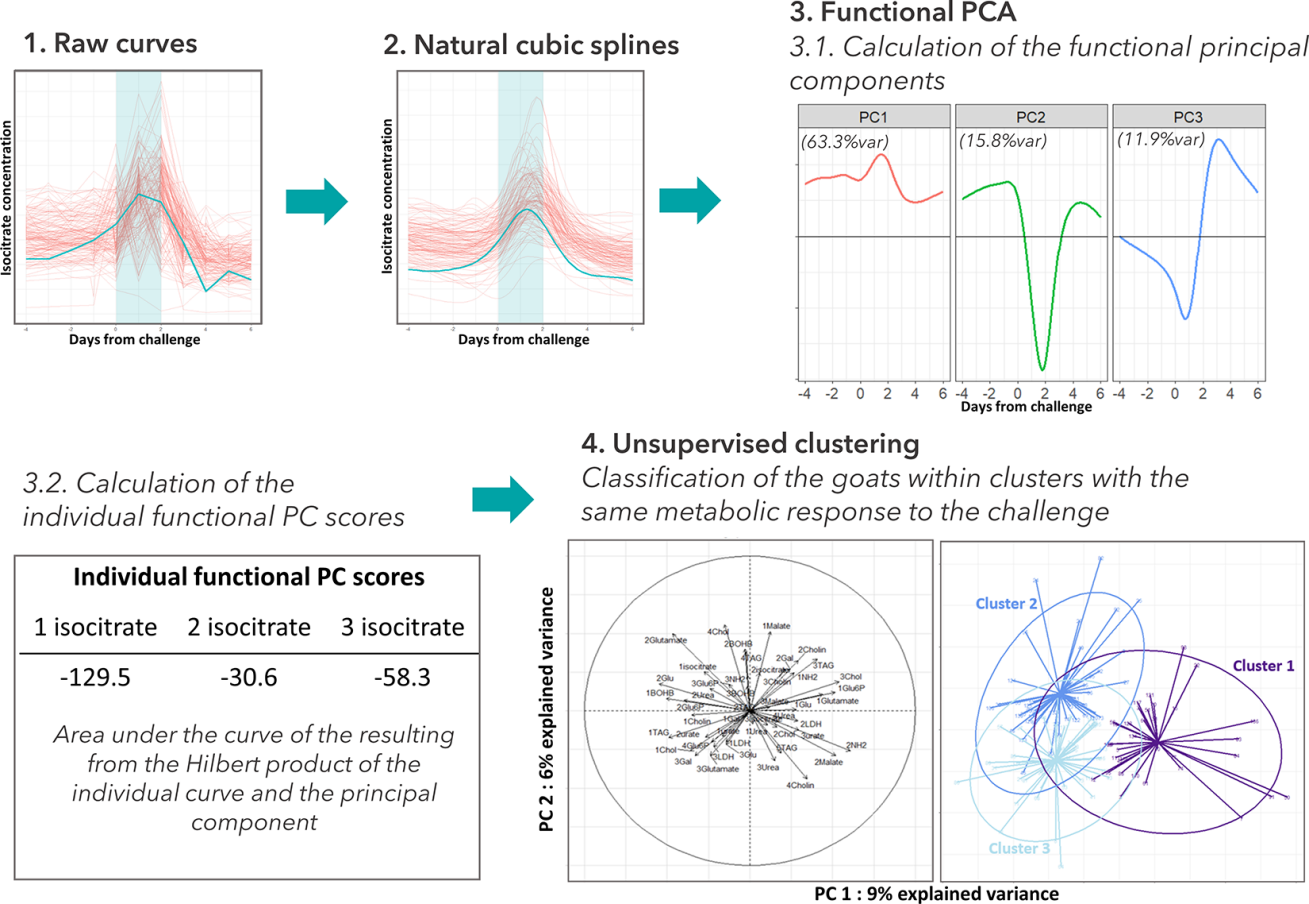
Scheme showing the different stages of analysis of one milk metabolite curve dataset (here isocitrate is shown as example). In the raw curves plot (1) and smoothed curves plot (2), each red line corresponds to one goat. The bold blue line corresponds to one randomly chosen goat. The functional principal components (PC) of the functional principal components analysis (fPCA) for isocitrate are plotted in (3.1), and the corresponding scores for the randomly chosen goat are shown in (3.2). The fPCA scores of the 13 milk metabolites and 1 enzyme are then used to classify the goats within 3 clusters with the same overall metabolic response to underfeeding challenge (4). Adapted from Ithurbide et al. (2023).

The types of data needed for nuanced phenotyping of resilience have not been traditionally easy to obtain. However, the increasing sophistication of on-farm precision livestock technologies makes it increasingly possible to achieve large-scale phenotyping for resilience. This is in terms of having time-series measurements but also in terms of having physiological measures such as metabolites, enzymes, and hormones. New statistical models have been, and will continue to be, developed for the integration of multivariate time series, to develop new resilience phenotypes that capture the underlying mechanisms of resilience. The recent studies reviewed here have shown that operational and heritable resilience proxies exist, that they can form the basis for selection for resilience, and that more nuanced phenotypes are attainable that will allow selection for resilience to be tailored according to prevailing environmental challenge types.
